# Risk of Poor Outcomes with COVID-19 Among U.S. Detained Immigrants: A Cross-Sectional Study

**DOI:** 10.1007/s10903-021-01173-z

**Published:** 2021-03-04

**Authors:** Caitlin Patler, Altaf Saadi

**Affiliations:** 1 Department of Sociology, University of California Davis, 1283 Social Sciences & Humanities, One Shields Ave, Davis, CA 95616 USA; 2 Department of Neurology, Massachusetts General Hospital, Harvard Medical School, Boston, MA USA

**Keywords:** COVID-19, Immigration, Unauthorized immigrants, Detention

## Abstract

Conditions in immigrant detention centers facilitate the spread of infectious diseases like COVID-19. However, there is no publicly-available data on detainees’ health characteristics, making it difficult to estimate the prevalence of risk among detained people. We use cross-sectional survey data from the only survey of detained immigrants, conducted in California in 2013–2014, to assess the prevalence and health-related correlates of health conditions among detained immigrants. We calculated the proportion of detained immigrants with chronic conditions, their interruptions in care, and stratified by sociodemographic characteristics, evaluating differences using two-tailed tests. Among 529 detained immigrants, 42.5% had at least one chronic health condition; 15.5% had multiple chronic conditions. 20.9% experienced disruption in care upon entering detention. 95.6% had access to stable housing in the U.S. Many detained people face health conditions that confer greater risk for poor outcomes with COVID-19. Stable residence can facilitate release of detainees via Alternatives to Detention programs.

## Introduction

U.S. Immigration and Customs Enforcement (ICE) detains hundreds of thousands of immigrants per year, across hundreds of detention facilities, the majority operated by for-profit prison corporations. Extensive previous documentation reveals that detention conditions are conducive to the rapid spread of infectious disease through overcrowding, poor ventilation, inadequate sanitation and healthcare services provision, and contact with staff across multiple shifts each day [1, 2]. Prior infectious disease outbreaks have included mumps, measles, and influenza [3, 4].

Carlos Ernesto Escobar Mejia became the first person in ICE custody to die from COVID-19 on May 6, 2020. Health and legal professionals have raised alarm that many detainees may be similarly imperiled by COVID-19 infection given the conditions of confinement in carceral settings [5]. From February 2020 to August 10, 2020, ICE reported 4444 total confirmed cases of COVID-19 within its detention facilities, from a total of 22,580 [6], yielding a positive rate of nearly 20%. Increases in COVID-19 testing have not fully explained increasing monthly case rates during the pandemic, suggesting limitations in ICE’s mitigation efforts [7].

Yet there is no comprehensive, publicly-available information on the clinical characteristics of detained immigrants that could help assess the prevalence of individuals who may be at higher risk for severe illness and death from COVID-19, according to the Centers for Disease Control and Prevention (CDC) [8]. We analyze the only systematic cross-sectional health survey of detained adult immigrants in California to demonstrate the prevalence of chronic conditions, interruptions in healthcare, and sociodemographic characteristics of detained people who may be at risk for poor outcomes with COVID-19.

Understanding detained immigrants’ health conditions is vital to decision-making on the part of policymakers, public health professionals, legal advocates, and immigration enforcement agencies to mitigate virus spread, reduce morbidity and mortality among detained immigrants, and minimize impact on local healthcare resources.

## Methods

### Design

This is a cross-sectional secondary analysis of baseline data from the Rodriguez Survey (RS), the only survey of detained people to ask health-related questions, including medical diagnoses and treatment history [9].

### Participants and Setting

Participants were drawn from a census of individuals detained in the Central Federal Court District of California who were class members of *Rodriguez v. Robbins*, a class action litigation establishing the right to bond hearings for detainees held beyond 180 days (715 F.3d 1127 (9th Cir. 2013). Study participants were drawn from a census of individuals who had been scheduled *Rodriguez* bond hearings; the response rate was 92 percent. Surveys took place in four facilities: three county or city jails and one facility operated by a for-profit correctional corporation. These facilities housed all *Rodriguez v. Robbins* class members during the study period (2013–2014).

### Data Collection

A 92-question in-person survey was administered in English or Spanish to 565 detainees who were at least 18 years old (response rate, 92%). The survey was conducted in partnership with a legal organization to facilitate access to the population. Survey materials were adapted from research on incarceration and customized for noncitizens detained under immigration law [9]. The survey captured detailed information about respondents’ legal history, health, and sociodemographic backgrounds. Surveys lasted between 90 and 120 min and respondents did not receive compensation for participating. Analysis of de-identified study data received approval by the first author’s Institutional Review Board.

### Data Analysis

We restricted analysis to participants with non-missing data on any of the study variables (n = 529). We calculated the proportion of detained immigrants with risk factors for poor outcomes with COVID-19, per CDC classifications [8], including one or more chronic conditions. To capture health care delivery challenges in the detention context, we also examined interruptions in care upon detention which could create additional vulnerability for detainees. Finally, we stratified the sample by self-reported sociodemographic characteristics that may be associated with health due to systemic structural inequalities, evaluating differences in proportions and means using two-tailed tests with a 2-sided p-value of less than 0.05 for significance to compare respondents. We conducted all analyses using Stata 16.1.

## Results

Table 1 shows that the mean age of participants (N = 529) was 37.4 years (range = 18.6–68.9 years), with 0.1% older than 65. 91.9% were men and 91.1% were from Latin America (including Mexico, Central, and South America). Just under half (42.9%) reported having a high school degree, and 51.6% reported speaking English well or very well. 59.6% had access to some form of health insurance prior to detention. 95.6% had access to stable housing in the United States.

**Table 1:** Sociodemographic characteristics of detained adult immigrants in the rodriguez survey

Sociodemographic characteristic	Total sample	Any chronic condition	Multiple chronic conditions (MCC)
	N/529 (%) or mean (range)	N/225 (%) or mean (range)	N/82 (%) or mean (range)
Gender: male (vs. female)	486 (91.9)	205 (91.1)	71 (86.6)
Age (Years)^a^	37.4 (18.6–68.9)	40.0 (19.3–68.9)*	41.6 (21.6–68.9)*
65 or Older	3 (0.1)	2 (0.9)	2 (2.4)
Region of Origin: Mexico, Central America, South America (vs. other regions)	482 (91.1)	191 (84.9)*	68 (82.9)*
High school degree or more	227 (42.9)	112 (49.8)*	40 (48.8)
Speaks English very well/well (vs. just a little/not at all)	273 (51.6)	130 (57.8)*	54 (19.8)*
Pre-detention health insurance	315 (59.6)	111 (49.3)*	35 (42.7)*
Access to stable housing in the U.S.^c^	515 (97.4)	215 (95.6)*	77 (93.9)*

^a^Age calculated at date of survey date and then stratified to identify individuals aged 65 and older, per Centers for Disease Control and Prevention

^b^Health insurance = employer-covered, Medicare, MediCal/Medicaid, VA health care, covered through family, other; vs. No insurance

^c^Calculated by pooling individuals who lived in own/family/friends/relative home prior to detention, compared to those who were homeless or living in residential treatment facilities, transitional housing, shelter, motel/hotel, or reported no set place[to live]/moved around a lot

^*^p < *.05* (χ^2^ for proportions; two-sided t-tests for averages)

We find that 42.5% of respondents had at least one diagnosed medical condition and 15.5% had multiple chronic conditions (Table 2). The most common conditions were metabolic syndrome (27.8%), neuropsychiatric conditions (16.7%), and lung disease (8.9%). Of individuals with health conditions and known treatment information, 20.9% experienced disruption in care upon entering detention, as did 31.9% of individuals with two or more conditions.

**Table 2:** Medical conditions & continuity of care among detained immigrants

Diagnosed health condition	RS total sample	Interrupted care, of those with condition ^a^
	Frequency/529 (%)	Proportion (%)
Any condition below	225 (42.5)	44/210^b^ (20.9)
Multiple (2 +) chronic conditions below	82 (15.5)	23/72^b^ (31.9)
Lung Disease	47 (8.9)	18/43^b^ (41.9)
Chronic lung illness	8 (1.5)	4/7^b^ (57.1)
Asthma	31 (5.9)	8/30^b^ (26.7)
Tuberculosis	19 (3.6)	9/17^b^ (52.9)
Cardiovascular disease	29 (5.5)	5/29 (17.2)
Heart trouble, heart disease, angina	25 (4.7)	3/25 (12.0)
Stroke	5 (1.0)	2/5 (40.0)
Immunocompromised status	15 (2.8)	2/15 (13.3)
Cancer	3 (0.1)	2/3 (66.7)
HIV or AIDS	13 (2.5)	0/13 (0)
Metabolic syndrome	147 (27.8)	16/145^b^ (11.0)
High blood pressure or hypertension	102 (19.3)	4/102 (3.9)
Diabetes	34 (6.4)	2/34 (5.9)
High cholesterol/triglycerides	79 (14.9)	10/77^b^ (13.0)
Liver Disease	14 (2.7)	1/14 (7.1)
Neuropsychiatric condition	89 (16.8)	13/78^b^ (16.7)
Depression	78 (14.7)	9/72^b^ (12.5)
Mental health condition other than depression	38 (7.2)	4/34^b^ (11.8)
Learning disability or cognitive disorder	15 (2.8)	2/12^b^ (16.7)

^a^Respondents receiving care prior to detention who no longer received care during detention for that health condition

^b^Denominator varies from numerator in total sample due to missing data on treatment before and/or during detention

Several groups of detainees are overrepresented among those with any health condition and with multiple chronic conditions, including individuals who are: older, not from Latin America, speak English better, without health insurance prior to detention, and without access to stable housing in the United States (Table 1).

## Discussion

Although detained immigrants are relatively young, nearly 43% of study respondents had at least one chronic medical condition and nearly 16% had multiple chronic conditions that could place them at high risk for severe illness or death from COVID-19. Even one chronic condition can increase risk for severe consequences from COVID-19; one study of COVID-19 patients revealed that more than 80% had more than one underlying medical condition [10]. These risks are heightened if health conditions are not adequately managed, e.g. through disruptions of medical care as described in our study. Receiving adequate COVID-19 care within the strained medical infrastructure in detention facilities may be especially challenging for detained people with chronic medical conditions [1].

While unique, our study is not without limitations. Our analysis of health conditions is limited to self-reported data and does not directly identify all currently-known CDC high-risk categories (such as morbid obesity, kidney disease, or smoking), or the severity of chronic conditions. The data also came from one geographic area, which may not represent all currently detained people, and were not gathered during the current pandemic. Despite these limitations, as the only systematic survey of detained immigrants’ health in the U.S., these data provide a critical foundation for understanding detainees’ health profiles.

COVID-19 cases among detained immigrants have continued to rise since Carlos Escobar Mejia’s death. Plans to mitigate COVID-19 spread should rely on evidence-based practices, including meticulous sanitation, hygiene, and testing. Particularly for those with one or more chronic conditions, decision-makers must consider every available option to mandate release from the congregate setting of detention centers in which social distancing is almost impossible even under ideal conditions. Release can be easily facilitated through existing Alternatives to Detention (ATD) programs in which individuals can be released to their families and communities as they continue with their immigration legal proceedings. Our study finds that the vast majority of detained immigrants having access to housing in the U.S., which facilitates such release, meeting both legal standards for release from detention and public health standards for social distancing. Efforts to ensure detained immigrants’ safety—including release via well-established ATD programs—must be urgently implemented.
